# A novel variant of COL6A3 c.6817-2(IVS27)A>G causing Bethlem myopathy: A case report

**DOI:** 10.3389/fneur.2023.1063090

**Published:** 2023-01-27

**Authors:** Maohua Li, Jiandi Huang, Min Liu, Chunmei Duan, Hong Guo, Xiaoyan Chen, Yue Wang

**Affiliations:** ^1^Department of Neurology, The Second Affiliated Hospital (Xinqiao Hospital), Army Medical University (Third Military Medical University), Chongqing, China; ^2^Department of Medical Genetics, College of Basic Medical Science, Army Medical University (Third Military Medical University), Chongqing, China

**Keywords:** Bethlem myopathy, COL6A3, muscular dystrophy, muscle MRI, COL6-RD

## Abstract

Bethlem myopathy (BM) is a disease that is caused by mutations in the collagen VI genes. It is a mildly progressive disease characterized by proximal muscle weakness and contracture of the fingers, the wrist, the elbow, and the ankle. BM is an autosomal dominant inheritance that is mainly caused by dominant COL6A1, COL6A2, or COL6A3 mutations. However, a few cases of collagen VI mutations with bilateral facial weakness and Beevor's sign have also been reported. This study presents a 50-year-old female patient with symptoms of facial weakness beginning in childhood and with the slow progression of the disease with age. At the age of 30 years, the patient presented with asymmetrical proximal muscle weakness, and the neurological examination revealed bilateral facial weakness and a positive Beevor's sign. Phosphocreatine kinase was slightly elevated with electromyography showing myopathic changes and magnetic resonance imaging (MRI) of the lower limb muscles showing the muscle MRI associated with collagen VI (COL6)-related myopathy (COL6-RM). The whole-genome sequencing technology identified the heterozygous mutation c.6817-2(IVS27)A>G in the COL6A3 gene, which was in itself a novel mutation. The present study reports yet another case of BM, which is caused by the recessive COL6A3 intron variation, widening the clinical spectrum and genetic heterogeneity of BM.

## Introduction

Bethlem myopathy (BM) was reported first by Bethlem and Wijngaarden (1). The onset of symptoms of BM usually occurs in early childhood, but occasionally in adulthood. The main symptoms of a patient with BM are the weakness of the proximal muscles and contracture of the fingers, the wrist, the elbow, and the ankle. Nevertheless, the presentation of BM is quite variable, and either contractures or muscular weakness may be absent (2). Its benign and slowly progressive course may lead to misdiagnosis. Recent studies showed magnetic resonance imaging (MRI) to be a supportive tool to diagnose collagen VI-related myopathies. Fu found that the “sandwich” sign in the vastus lateralis (VL) and the “target” sign in the rectus femoris (RF) were common in patients with collagen VI-related myopathies and were specific to these conditions (3). These MRI changes showed a positive predictive value of 69% for BM (4).

Herein, this study describes a 50-year-old female patient presenting with a slow progression of muscle symptoms, including proximal limb and facial weakness, since childhood. Finally, the diagnoses of BM in the patient in this study were confirmed genetically.

## Case presentation

**Figure 1:**
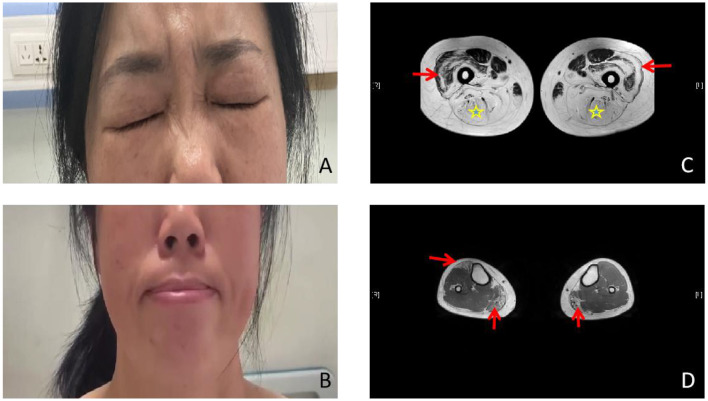
Photographs and lower limb muscle magnetic resonance imaging (MRI). **(A, B)** Photographs of the proband. Weakness of the eye and lip muscles. **(C)** The bilateral posterior thigh groups were markedly enlarged (star), especially of the lateral femoral muscle edges and central preservation (arrow). **(D)** Significant enlargement of the medial head of the gastrocnemius (arrow ↑) and the anterior tibial muscles in both lower legs.

The patient was a 50-year-old woman. She had no family history relevant to muscle weakness and was not from a consanguineous marriage. At the age of 5 years, she was unable to blow up balloons and gradually appeared to be unable to whistle or puff her cheeks. She had normal mental growth, and the progression of her disease became slower. At the age of 30 years, she presented with asymmetrical proximal muscle weakness in the lower limbs and milder asymmetrical proximal muscle weakness in the upper limbs, as well as mild weakness in the waist. The progression of muscle weakness was slow. She was unable to climb stairs, jump, run, and rise from the floor, but she had no difficulty in breathing. Her cardiovascular and respiratory examinations were normal. The bilateral eyelash sign was positive, and the right nasolabial fold was shallower than the left one. She was unable to grimace and puff her cheeks (Figures 1A, B). Mental function and other cranial nerve function tests showed normal values. Muscle weakness in the limbs was asymmetrical (Medical Research Council (MRC). Grades 4/5 proximally and 5/5 distally, and bilateral hip extensors 2/5) without limb muscle atrophy. Beevor's sign was positive. Her sensations were normal, and muscle stretch reflexes were not elicited.

**Figure 2:**
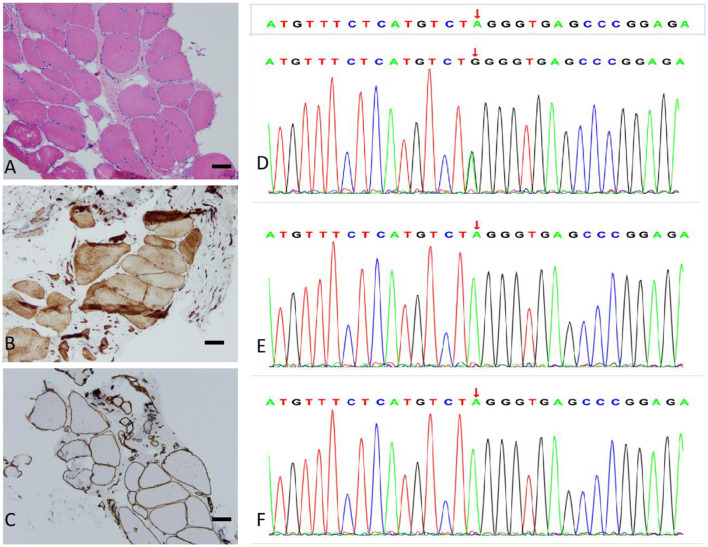
Muscle biopsy (bar = 100 μm) and genotype results. **(A)** Hematoxylin and eosin (H&E) staining: the skeletal muscle tissue of the right anterior tibial muscle exhibited a slight myogenic damage. **(B)** Dysferlin staining: the myofiber membrane and cytoplasm were positive. **(C)** α-Sarcoglycan staining: the muscle fiber membrane was positive, with a uniform and continuous expression. **(D–F)** Sequencing of the COL6A3 gene in the patient **(D)**, patient's mother **(E)**, and patient's son **(F)**. The variation c.6817-2(IVS27)A>G is present only in the patient.

Laboratory studies revealed a slightly elevated serum creatine kinase (CK) value of 291.5 international units per liter (IU/L; 26–174 IU/L). Respiratory parameters and cardiac evaluation [echocardiogram (echo) and electrocardiogram (ECG)] were unremarkable. Electromyography (EMG) showed myopathic changes, and nerve conduction was normal. The lower limb muscle MRI showed that the bilateral posterior thigh groups were markedly fattened, especially of the lateral femoral muscle edges and central preservation. A significant enlargement of the medial head of the gastrocnemius and anterior tibial muscles was observed in both lower legs (Figures 1C, D). Muscular pathological findings showed that the right tibial premuscle muscle fibers were clearly unequal in size, some of the muscle fibers were more clearly atrophied, some of the muscle fibers were mildly hypertrophied, a few muscle fiber nuclei were internally displaced, individual muscle clefts and nuclei aggregation were seen, muscle fiber degeneration and necrosis were not visible, and collagen VI immunostaining revealed a normal staining pattern (not shown; Figures 2A–C). Molecular studies, using the whole-genome sequencing technology, identified the heterozygous mutation c.6817-2(IVS27)A>G in the COL6A3 gene (Figures 2D–F). Sanger sequencing confirmed that the mutation was not detected in her mother or her son. Coenzyme Q10, inosine tablets, and vitamin E were administered to the patient. During hospitalization, rehabilitation doctors gave her rehabilitation training. After discharge, the patient was instructed to avoid strenuous exercise but to continue appropriate stretching, strength training, and muscle massage. Despite nerve nutrition and rehabilitation physiotherapy, there was no significant improvement in the patient's condition.

## Discussion

Collagen VI is a ubiquitous extracellular matrix protein that forms a microfibrous network. It is closely related to BM, Ullrich congenital muscular dystrophy (UCMD), and autosomal recessive myosclerosis myopathy. It is also closely associated with the basement membrane in the muscle, the cartilage, the periosteum, the ligaments, the cornea, the skin, the tendon, and the bone (5). Collagen VI consists of three different peptide chains: α1, α2, and α3. Each peptide chain has a triple helical (TH) region containing a single cysteine residue, with the defining Gly-X-Y amino acid sequence flanked by extensive N- and C-terminal globular domains that have subdomains with homology to the type A domains of the von Willebrand factor (6). The α3 (VI) chain has three additional C-terminal domains, a unique region (C3), a fibronectin type III repeat (C4), and a Kunitz-type protease inhibitor motif (C5), which can be cleaved extracellularly. The TH region folds in a zipper-like fashion from the C- to N-terminus to form a triple helix monomer and then assembles further into a tetramer. These tetramers are secreted into the extracellular matrix, where they assemble in an end-to-end fashion to form a microfiber network. The deficiency of collagen VI leads to an increase in apoptosis and oxidative stress, a decrease in autophagy, and an impairment of muscle regeneration (7). The genetics of collagen VI-related dystrophies (COL6-RD) is complex. COL6-RD is a disease entity that is caused by both dominant and recessive mutations in the three collagen VI-related genes, COL6A1, COL6A2, and COL6A3 (8). Patients with COL6-RD show a unique pattern of muscle involvement in MRI, which is an inward (outside-in) progression [starting from the fascia planes and progressing into the vastus lateralis (VL), the lateral gastrocnemius (LG), and central shadowing in the rectus femoris (RF)] of the fiber-fat material based on T1-weighted images. This inward progression of fiber-fat conversion causes the formation of a lipid-rich structural ring around the muscle whose thickness increases with an increasing severity of disease, and the muscle tissue is replaced by the fiber-fat material as the disease advances (9). The most common mutation in collagen VI myopathy affects the conserved Gly-X-Y motif in the TH domain (10).

The α3 chain is encoded by the COL6A3 gene and plays a determinant role in the monomer assembly. Mutations in the COL6A3 gene have been associated with UCMD and BM. Biallelic variants in COL6A3 have recently been suggested to be the cause of an early-onset isolated dystonia syndrome (DYT27) (11). Due to remarkable heterogeneity of the COL6 genes at the clinical and molecular levels, it is difficult to explain the clinical symptoms of the COL6A3 variants. The COL6A3 c.7447A>G variant in a homozygous state can lead to a mild Bethlem myopathy and/or the limb-girdle muscular dystrophy (LGMD) phenotype without respiratory involvement; however, a compound heterozygous mutation exhibits phenotypes ranging from a mild phenotype to an intermediate phenotype and a severe Ullrich-like phenotype (10). It has been reported that, in patients with other mutations in a compound heterozygous state, the phenotype severity depends entirely on the second mutation (10).

The present study reported a 50-year-old woman who had proximal limb weakness, bilateral facial weakness, and a positive Beevor's sign. Her CK was mildly elevated, and an electromyographic study revealed myopathic changes. The present study considered first facioscapulohumeral muscular dystrophy (FSHD), an inherited muscle disease that is characterized by progressive atrophy and weakness of the facial, shoulder limb-girdle, abdominal, and anterior leg muscles. FSHD has a tendency toward atypical findings, so it may be confused with other neuromuscular diseases. Muscle biopsy of FSHD most often shows non-specific chronic myopathic changes (12). Muscle MRI is a reliable tool to differentiate FSHD from other muscular dystrophies. Patients with FSHD exhibit a widespread involvement of the lower limb muscles, particularly the hamstring and the posterior calf muscles (13). However, in this patient, the bilateral posterior thigh groups were markedly enlarged, especially of the lateral femoral muscle edges and central preservation. Significant enlargement of the medial head of the gastrocnemius and anterior tibial muscles in both the lower legs was associated with COL6-RD-like muscles on MRI (4). Ultimately, the next-generation whole-genome sequencing revealed an intronic mutation in COL6A3, c.6817-2(IVS27)A>G. The heterozygous c.6817-2(IVS27)A>G mutation in the splice site of intron 27 led to exon skipping. The mutation was not present either in the unaffected mother or in the son, thereby indicating that it was a novel mutation that was present only in the patient. This mutation has also not been recorded in the Single Nucleotide Polymorphism Database (dsSNP) database, the Exome Aggregation Consortium (EXAC) database, or the 1,000 Genomes Project (1 KGP) database. According to the American College of Medical Genetics and Genomics (ACMG) standards and guidelines, the variant was classified as a likely pathogenic variant [very strong evidence of the pathogenicity criterion (PVS1)+absent from controls (PM2)]. Because the patient's father has died, the patient refused to undergo Southern blotting or molecular combing and her sisters also refused to undergo the whole-genome sequencing; therefore, it was impossible to identify cosegregation with the phenotype within the family. Five patients mentioned in the report by Lee et al. (8) showed bilateral facial weakness, and all but one patient did show limb weakness. Therefore, the patient was diagnosed with probable BM (OMIM: 158810) based on her medical history, clinical examinations, muscle MRI, and genetic test results.

## Conclusion

In conclusion, the present study describes a patient carrying a heterozygous COL6A3 c.6817-2(IVS27)A>G pathogenic splicing variant. The patient presented, since childhood, with facial weakness associated with proximal muscles, and the muscle MRI showed COL6-RM. Further analysis of molecular and genetic studies would help to elucidate whether this variant is of the disease-causing type. The muscle immunohistochemical marking for the COL6 protein is variable (14) and cannot be used as the basis for excluding the disease, while muscle MRI and genetic test are helpful in diagnosis. Currently, there are no pharmacological disease-modifying treatments for collagen VI-congenital muscular dystrophy (COL6-CMD). Genetic counseling and prenatal diagnosis are particularly important.

## Data availability statement

The datasets presented in this article are not readily available because of ethical and privacy restrictions. Requests to access the datasets should be directed to the corresponding authors.

## Ethics statement

Written informed consent was obtained from the individual(s) for the publication of any potentially identifiable images or data included in this article.

## Author contributions

MaL and JH wrote the manuscript draft. MiL, HG, and CD planned, designed, and analyzed the data. XC and YW organized and proofread the writing of the manuscript. All authors contributed to the article and approved the submitted version.
